# A Full GMP Process to Select and Amplify Epitope-Specific T Lymphocytes for Adoptive Immunotherapy of Metastatic Melanoma

**DOI:** 10.1155/2013/932318

**Published:** 2013-09-30

**Authors:** N. Labarriere, A. Fortun, A. Bellec, A. Khammari, B. Dreno, S. Saïagh, F. Lang

**Affiliations:** ^1^Inserm, U892, 44000 Nantes, France; ^2^Université de Nantes, 44000 Nantes, France; ^3^CNRS, UMR 6299, 44000 Nantes, France; ^4^Unit of Cell and Gene Therapy, CHU Nantes, 44000 Nantes, France; ^5^Unit of Skin Cancer, CHU Nantes, 44000 Nantes, France

## Abstract

A number of trials of adoptive transfer of tumor-specific T lymphocytes have been performed in the last 20 years in metastatic melanoma, with increasingly encouraging results as the relevant melanoma antigens were identified and the purity/specificity of injected T cells improved. We have previously described a sorting method of epitope-specific T lymphocytes that uses magnetic beads coated with HLA/peptide complexes and we suggested that this method could be applied to a clinical setting. In the present work, we provide a detailed description of the whole GMP process of sorting and amplification of clinical grade T cells specific for the melanoma antigens Melan-A and MELOE-1. All the reagents used in this process including the sorting reagent were produced in GMP conditions and we document the optimization of the different steps of the process such as peptide stimulation, sorting, and amplification. The optimized procedure, validated in 3 blank runs in a clinical setting, allowed the production of at least 10^8^ pure (>90%) Melan-A- and MELOE-1-specific T cells within 28 days starting with 100 mL of blood from metastatic melanoma patients. This GMP process is thus ready to be used in an upcoming phase I/II clinical trial on metastatic melanoma patients.

## 1. Introduction

In cancer, the best argument in favor of adoptive cell transfer (ACT) is the demonstration that it can elicit clinical regressions of cancers not curable by other treatments. Initially established for hematopoietic tumors in an allogeneic setting, the beneficial effect of ACT has also been documented in autologous situations such as the control of EBV-induced tumors by virus-antigen-specific T cells [1]. In metastatic stage III (AJCC 2010) melanoma patients, we have documented the beneficial effect on both relapse free survival and overall survival of adoptive transfer of *in vitro* amplified tumor-infiltrating lymphocytes (TIL), suggesting that tumor-reactive T cell transfer may be an efficient treatment in melanoma when performed at an early stage of the disease [2–4]. In advanced stage of melanoma, the clinical efficacy of ACT needs to be further improved. Indeed, although we and others have documented tumor regressions after the ACT of highly selected TIL or melanoma-specific cytotoxic T lymphocytes (CTL) clones in stage IV metastatic melanoma patients [5–7], clinical results are far from optimal. This suboptimal efficiency could be due to the selection of a single T cell clone that turns out to be poorly active *in vivo* and to a possible exhaustion of infused T cells, due to multiple steps of cloning and *in vitro* expansion, leading to a weak persistence *in vivo*. Ideal cells for ACT trials should combine specificity, polyclonality, tumor reactivity, and a potential long-term persistence *in vivo*. Thus, we consider that the efficacy of ACT could be improved by the infusion of polyclonal T cells, fully specific for highly immunogenic antigens, and produced through a relatively short process. Among the many melanoma antigens that have been identified so far, we chose to target the melanocytic differentiation antigen Melan-A/MART-1 and the melanoma overexpressed antigen MELOE-1. Indeed, (i) these two antigens are very frequently expressed by melanomas [8, 9], (ii) two immunogenic peptides derived from these two antigens are recognized by melanoma-specific T cells in the HLA-A2 context [10, 11], (iii) a broad and diverse T cell repertoire specific for these two epitopes is present in all HLA-A2 melanoma patients [12–14], and (iv) CD8 T lymphocytes specific for these two epitopes seem to be involved in melanoma immunosurveillance [9, 15]. Moreover, the advantage of targeting two antigens simultaneously will be to limit the selection of epitope-loss tumor variants, a major tumor escape mechanism that happens when a single epitope is targeted.

To select and expand such specific T cells, we developed an original method of T cell sorting from blood sample, based on the selection of specific T cells with HLA-peptide coated magnetic beads [16, 17]. This procedure, followed by a classical amplification step on irradiated feeder cells, allows the production of at least 10^8^ T cells specific for each of the targeted epitopes in about 32 days as compared to the four months needed to produce antigen-specific T-cell clones. Recovered T cells are polyclonal, specific (at least 90% pure), and reactive against HLA-A2 melanoma cell lines expressing the target antigens. 

To adapt this procedure for a clinical use, this requires that the cell production facility, the ancillary products, and the production process used meet the GMP (Good Manufacturing Practices) criteria in terms of quality controls (characterization and purity of produced T cells), safety (absence of microbiological contamination), and robustness (reproducibility and repeatability of the production process). 

To this aim, in collaboration with an industrial partner, PX'Therapeutics, we produced clinical grade batches of HLA-peptide-coated magnetic beads (Clinimers) in order to treat seventeen HLA-A2 metastatic melanoma patients at stage IIIc (not operable lymph node metastases) or IV (distant metastases) in an upcoming clinical trial. Starting from 100 mL of blood, Melan-A- and MELOE-1-specific T cells will be selected and amplified *in vitro* for each patient, who will receive intravenously a single infusion of at least 10^8^ cells of each specificity, associated with low doses of IL-2. In the present report, we describe the optimization steps that led to a robust and reproducible GMP process to generate melanoma-specific effector T cells and the validation of the whole process in three dry runs performed in a dedicated structure.

## 2. Material and Methods

### 2.1. PBMC and Cell Lines

Blood was collected from healthy HLA-A2 donors (Etablissement Français du Sang (EFS), Nantes, France) or from metastatic melanoma patients (Unit of Skin Cancer, Centre Hospitalier Universitaire Hotel Dieu, Nantes) after written informed consent. 

The two melanoma cell lines M113 and M117 were established from metastatic tumor fragments in the Unit of Cell therapy of Nantes and are registered in the Biocollection PC-U892-NL (CHU Nantes).

### 2.2. Peptide Stimulation of PBMC

Peripheral blood mononuclear cells (PBMC) were isolated by Ficoll-Hypaque gradient centrifugation, washed three times, and seeded in 96 well/plates at 2 × 10^5^ cells/well in either RPMI 1640 medium supplemented with 8% human serum (HS) (a pool from 20 donors prepared and secured by the EFS of Nantes) or in X-Vivo 15 serum-free medium (Lonza, Levallois-Perret, France) with various concentrations of IL-2 (from 10 IU/mL to 150 IU/mL). PBMC were stimulated by adding various concentrations of clinical grade Melan-A_A27L_ peptide (ELAGIGILTV) or MELOE-1_36-44_ peptide (TLNDECWPA) ranging from 0.1 *μ*g/mL to 10 *μ*g/mL for 14 days. Following stimulation, each microculture (each well) was evaluated for the percentage of specific CD8 T lymphocytes by double staining with the relevant APC-conjugated HLA-peptide tetramer (from the SFR Sante recombinant protein facility) and PE-conjugated anti-CD8 mAb (BD Biosciences, France) using a FACSCalibur. Microcultures that contained at least 0.5% of Melan-A_A27L_- or MELOE-1_36-44_-specific T cells were selected, pooled, and sorted with the relevant Clinimers.

### 2.3. Preparation of the Sorting Reagent Clinimers

The design of HLA-peptide multimers that we used for specific T cell sorting was previously described [16] and we have adapted our technology to be suited to GMP production and to increase biosafety. In brief, clinical grade M450-epoxy magnetic beads (Clin *Ex-vivo* Dynabeads, Life Technologies, St-Aubin, France) were covalently coupled to a monoclonal antibody specific for the peptide AviTag (Avidity, Aurora, CO, USA) that is fused to the heavy chain of our HLA constructs. We modified the initial AvT-6A8 mAb produced from mouse hybridoma (European Patent no. 08775037.8) to produce a chimeric mAb containing the human IgG1 constant region, named Chim-AvT, that we produced in the clinical grade CHO-DG44 cell-line (Life technologies). A master cell bank was made and delivered to PX'Therapeutics to produce clinical batches of Chim-AvT mAb in their GMP facility. The clinical grade Chim-AvT beads remained stable for over 12 months when stored at 4°C in a solution of PBS containing 0.1% of human serum albumin (HSA) (Octapharma, Boulogne-Billancourt, France).

HLA-A0201/peptide *α*3-mutated monomers were generated as previously described [18] except that the original biotinylation sequence was replaced by the AviTag sequence (GLNDIFEAQKIEWHE) (Avidity). Recombinant proteins were produced in GMP conditions by PX'Therapeutics as inclusion bodies in *E. coli*, dissolved in 8 M urea, and refolded with either clinical grade Melan-A_A27L_ peptide (ELAGIGILTV) or MELOE-1_36-44_ peptide (TLNDECWPA) (C S Bio, Menlo Park, CA, USA). HLA/peptide monomers were then purified by gel filtration and ion exchange in GMP conditions by PX'Therapeutics.

Final assembly of the Clinimer reagent was performed immediately before T cell sorting by incubating Chim-AvT beads with the appropriate HLA/peptide monomers (1 *μ*g of monomer for 10^7^ beads) for 1 hr at room temperature on a rotating wheel in PBS 0.1% HSA followed by 5 washes on a DynaMag-2 magnet (Life technologies). The quality control of the resulting clinical grade multimers of HLA/peptide complexes (Clinimers) was performed by staining with a PE-conjugated anti-HLA-A2 mAb (BD Biosciences, France).

### 2.4. Sorting Procedure and Polyclonal Amplification

Sorting of Melan-A and MELOE-1-specific T cells was performed by mixing a T cell suspension (5 × 10^6^ to 10^7^ cells) containing at least 2 × 10^5^ specific T cells with Clinimers at a ratio of 1 bead per cell in PBS 4% HSA, as previously described [16]. After 4 h, at room temperature under rotation, nonspecific lymphocytes were removed by successive washes on a DynaMag-2 magnet. Clinimer-covered specific T cells (rosettes) were seeded at 1000 rosettes/well in 96-well plates for polyclonal amplification as previously described [2, 3], with irradiated feeder cells, clinical grade IL-2 (150 IU/mL) (Novartis Pharma, Rueil Malmaison, France), and PHA-L (1 *μ*g/mL) (Sigma, Lyon, France), in 150 *μ*L of culture medium. Feeder cells were a mix of irradiated PBMC from 3 donors that was secured and provided by EFS (10^5^ cells/well) and a secured EBV-transformed B cell-line LAZ (10^4^ cells/well) from which a Master cell bank was made in the Unit of Cell Therapy of Nantes.

T cell cultures are split in half every 2 to 3 days and fresh medium is added. After about 8 ± 1 days, cells are transferred to 6-well plates for a final bulk amplification of 7 days. This open culture system was chosen because of a better efficiency of T cell amplification than in dedicated cell bags (data not shown). At this step, 10^8^ lymphocytes are seeded at 5 × 10^5^ cells/mL in 5 mL of medium per well, containing 150 U/mL of IL-2. Every two to three days cell concentration is adjusted to 5 × 10^5^ cells/mL in the same medium, until day 14.

### 2.5. Functionality of Sorted T Cells

The specificity of the sorted and amplified T lymphocytes was evaluated by cytokine production in response to their cognate epitope. In brief, T cells were stimulated for 5 h in the presence of brefeldin A (10 *μ*g/mL) either with their cognate peptide (10 *μ*M for MELOE-1_36-44_ and 1 *μ*M for Melan-A_A27L_) in an autopresentation assay or with HLA-A2 melanoma cell lines (M113 and M117) naturally expressing both antigens. Cells were then stained with APC-conjugated tetramer, fixed with 4% paraformaldehyde (Euromedex), permeabilized with PBS 0.1% saponin, intracellularly labelled with PE-conjugated anti-TNF-*α* mAb (BD Biosciences) for 30 min at room temperature, and analyzed by flow cytometry.

## 3. Results and Discussion

### 3.1. Step 1: Preamplification of Antigen-Specific T Cells by Peptide Stimulation

**Figure 1:**
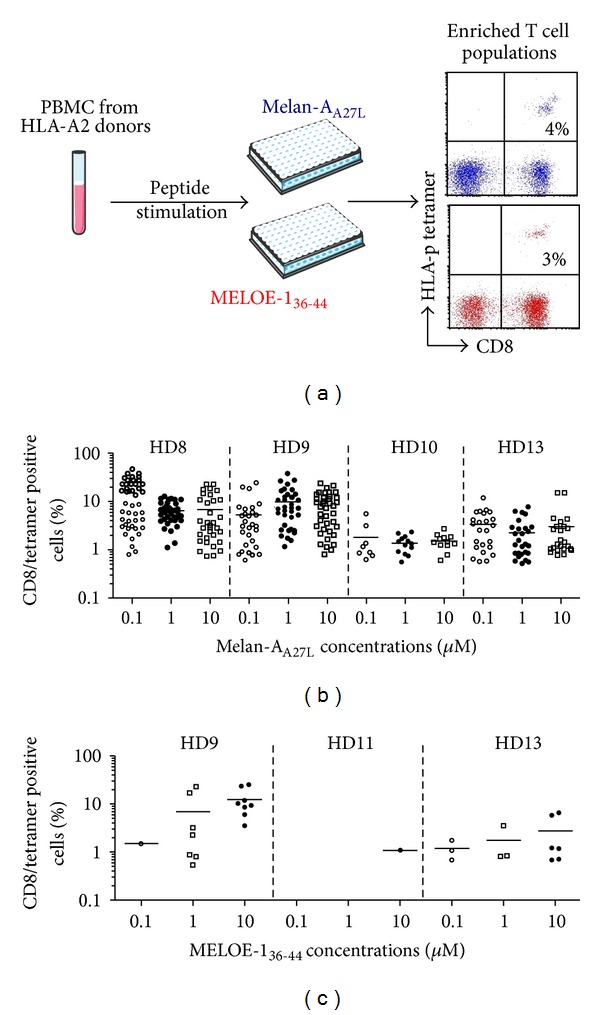
Peptide stimulation step. (a) 10^7^ PBMC from HLA-A2 healthy donors were stimulated in 96-well plated (containing 2 × 10^5^ cells/well) for 14 days with Melan-A or MELOE-1 peptides. (b) Percentages of Melan-A- or (c) MELOE-1-specific T cells detected by double labelling with tetramer and anti-CD8 antibody, in microcultures stimulated with 0.1 to 10 *μ*M of Melan-A_A27L_ or MELOE-1_36-44_. Each symbol corresponds to a microculture containing more than 0.5% of specific T cells.

We have previously demonstrated that to ensure efficient sorting of specific T cells with HLA multimers, that is, high yields and high purity (>90%) in all donors, the starting PBMC populations should contain at least 0.5% of specific T cells and thus a short peptide stimulation is required that does not alter T cell repertoire [17]. Therefore, the first step of the process was the enrichment of antigen-specific T cells among PBMC by specific peptide stimulation in culture medium containing IL-2, during 14 days (Figure 1(a)).

For each antigen, we first defined the optimal peptide concentration for amplification of Melan-A (Figure 1(b)) and MELOE-1 (Figure 1(c)) specific CD8+ T lymphocytes, detected by CD8/HLA-peptide (HLA-p) tetramer double staining. Concerning Melan-A-specific T cells, PBMC from 4 HLA-A2 healthy donors were stimulated in 48 microcultures with either 0.1, 1, or 10 *μ*M of Melan-A_A27L_ peptide. This decapeptide is modified at the P2 anchor position (A↔L), in order to enhance and stabilize the binding into the HLA-A2 molecule [19]. Furthermore, T cells stimulated with this modified peptide are able to recognize the natural epitope on HLA-A2^+^ melanoma cells [6, 20]. As shown in Figure 1(b), no significant difference was observed between these three peptide concentrations, neither in terms of number of positive microcultures, nor in terms of percentages of specific T lymphocytes within positive microcultures. The intermediate concentration of 1 *μ*M of Melan-A_A27L_, previously used to generate Melan-A-specific T-cell clones for adoptive transfer to HLA-A2 melanoma patients [5, 6, 20], was thus selected. Concerning MELOE-1, the number of positive microcultures was about ten fold lower than the number obtained after Melan-A stimulation, which was consistent with the differences in the breadth of Melan-A- *versus* MELOE-1-specific T cell repertoires observed in HLA-A2 healthy donors [12]. The optimal MELOE-1_36-44_ peptide concentration to amplify specific T cells was 10 *μ*M (Figure 1(c)), thereafter used in later experiments.

**Figure 2:**
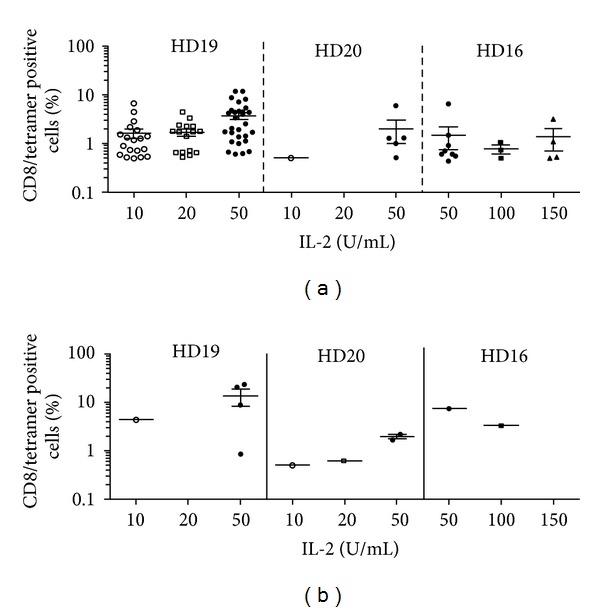
Influence of IL-2 concentration on the efficiency of peptide stimulation. 10^7^ PBMC from HLA-A2 healthy donors were stimulated in 96-well plated (2 × 10^5^ cells/well) with either Melan-A_A27L_ peptide (1 *μ*M) or the MELOE-1_36-44_ peptide (10 *μ*M). (a) Percentages of Melan-A- or (b) MELOE-1-specific T cells detected by double labelling with tetramer and anti-CD8 antibody, in microcultures stimulated in the presence of various IL-2 concentrations. Each symbol corresponds to a microculture containing more than 0.5% of specific T cells.

To favor the proliferation of specific T cells, IL-2 is added to the culture medium, during the peptide stimulation step. However, IL-2 is a double edge sword since it can also induce apoptosis by AICD [21] or favor the proliferation of NK cells [22] or CD25+ Treg [23]. Therefore, various IL-2 concentrations were tested to define the optimal conditions for amplification of antigen-specific T cells. Indeed, for the two peptides, the addition of 50 U/mL of IL-2 enhanced the amplification of antigen-specific T cells, as shown by the increased number of positive microcultures and the higher fraction of specific T cells, as compared with 10 and 20 U/mL (Figures 2(a) and 2(b)). In contrast, higher concentrations (100 and 150 U/mL) did not improve the number of positive microcultures or the percentages of Melan-A- or MELOE-1-specific T cells. Thus, the concentration of 50 U/mL of IL-2 was chosen for later experiments.

The use of human serum for the amplification of T cells requires the production of dedicated batches by a transfusion center and the validation of each batch for the absence of known viruses. Because it is rather cumbersome and expensive, alternate culture conditions were tested.

**Figure 3:**
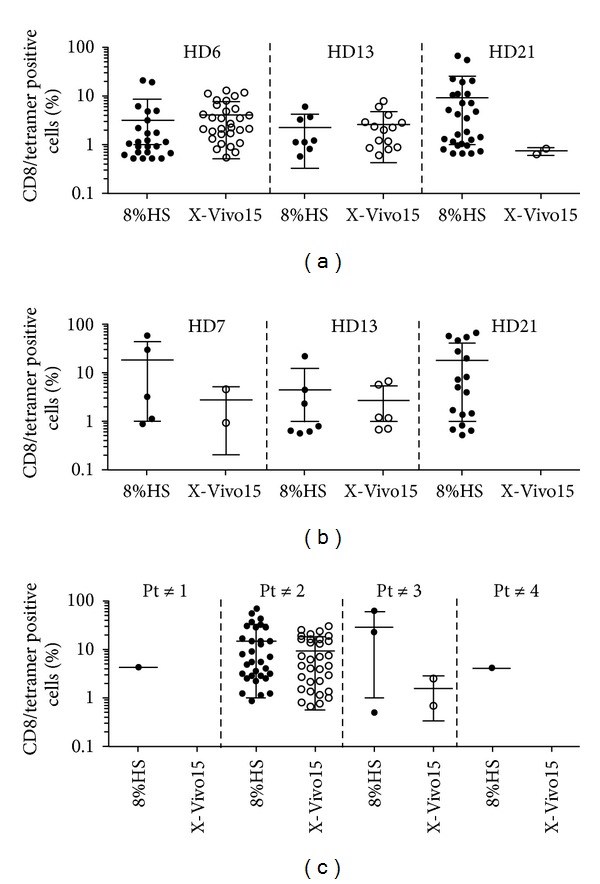
Influence of the culture medium on the efficiency of peptide stimulation. 10^7^ PBMC from HLA-A2 healthy donors (a and b) or from melanoma patients (c) were stimulated in 96-well plated (2 × 10^5^ cells/well) with either Melan-A_A27L_ peptide (a) at 1 *μ*M or MELOE-1_36-44_ peptide (b and c) at 10 *μ*M, in either RPMI 8% HS or X-Vivo 15 in the presence of 50 IU/mL of IL-2. (a) Percentages of Melan-A- or (b and c) MELOE-1-specific T cells detected by double labelling with tetramer and anti-CD8 antibody, in microcultures stimulated in the two culture media. Each symbol corresponds to a microculture containing more than 0.5% of specific T cells.

Since melanoma-derived TIL are successfully amplified in X-Vivo 15 medium [3], without any addition of HS, we tested this medium and compared it to RPMI 8% HS for the amplification of Melan-A- and MELOE-1-specific T cells, starting with PBMC from HLA-A2 healthy donors. As shown in Figures 3(a) and 3(b), for one donor (HD21) out of the three tested, X-Vivo 15 medium was much less favorable than RPMI 8% HS for the amplification of both Melan-A- and MELOE-1-specific T cells. This study was then extended to PBMC from HLA-A2 melanoma patients, concentrating on the amplification of MELOE-1-specific T cells, which represent the limiting population because of its narrower T cell repertoire [12]. Starting from 1.5 × 10^7^ PBMC from 4 patients (48 microcultures), at least one positive microculture was obtained for every patient after stimulation in RPMI 8% HS (Figure 3(c)) while the use of X-Vivo 15 allowed the amplification of MELOE-1 specific cells in only 2 out of 4 patients. Thus, the addition of human serum was critical for the amplification of antigen specific T cells when starting with low precursor frequencies, and we decided to use RPMI supplemented with 8% of HS in our production procedure.

### 3.2. Step 2: Coating of HLA-p Monomers on Antibody-Coated Beads and Sorting Procedure

**Figure 4:**
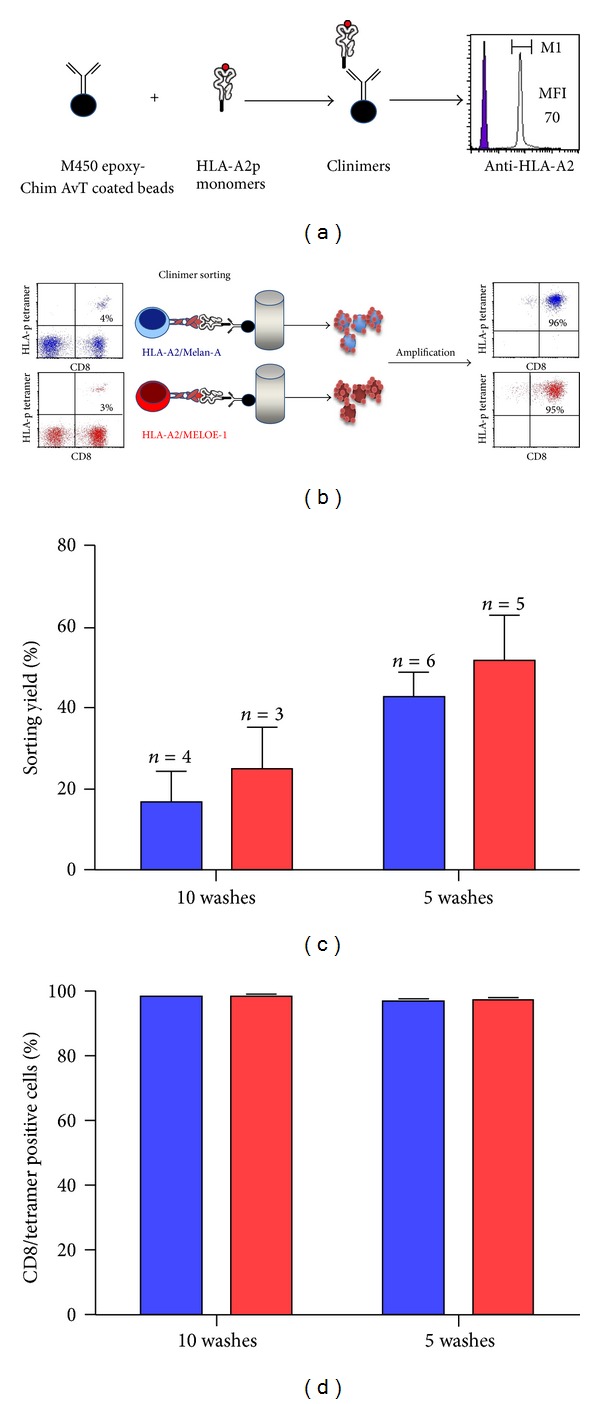
Sorting procedure. (a) 10^7^ Chim-AvT dynabeads are coated with HLA-A2-peptide monomers, and coating efficiency is assessed on 10^5^ beads, by labelling with an PE-labelled anti-HLA-A2 mAb (5 *μ*g/mL). (b) Peptide stimulated T cells, containing at least 1% of specific T cells are incubated with Clinimers (ratio 1/1) and recovered on a magnet. After washes in PBS, rosetted T cells are seeded in 96-well plates (10^3^ cells/well), containing irradiated allogeneic feeder cells, for amplification. After 14 days, the specificity of T cells is assessed by double labelling with an anti-CD8 mAb and each specific tetramer. (c) Influence of the number of washes on sorting yields. Sorting yields are calculated by dividing the number of rosetted T lymphocytes counted after the sort by the number of specific T cells in the sorted population estimated by tetramer staining. Blue bars represent Melan-A-specific T cells and red bars MELOE-1-specific ones. (d) Influence of the number of washes on purity of selected and amplified T cells. The purity of amplified sorted-T cells is assessed after the 14-day amplification period on feeder cells, by double staining with an anti-CD8 mAb and the specific tetramer. Blue bars represent Melan-A-specific T cells and red bars MELOE-1-specific ones.

As mentioned in Section 2, Chim-AvT coated beads are coated with HLA-A2-peptide monomers immediately before each sort, and the coating efficiency is checked by flow cytometry, using an HLA-A2 specific mAb. This procedure is illustrated in Figure 4(a). This coating step was very reproducible over time with an MFI (mean fluorescence intensity) calculated on 13 independent assays of 77 ± 14 with the same batch of Chim-Avt beads, over an eight-month period. This corresponds to an RFI (ratio of fluorescence intensity = MFI of HLA coated beads/MFI of noncoated beads) of 25 ± 3.

After the sorting step nonspecific lymphocytes are removed by successive washes on a magnet, which only retains Clinimers-coated T cells. These washes critically impact the yield and the purity of sorted T cells. Indeed, too many and/or too vigorous washes will result in Clinimers detaching from specific T cells and thus decrease the yield of the sort, while insufficient washing will lead to a lower purity due to nonspecific T cells that have not been discarded. In the sorting procedure that we initially described, 10 washes were recommended to ensure a high purity of sorted cells [16], but the yields were not our major concern at the time. However, when an adoptive transfer clinical trial is planned, yields become critical because they will have an impact on the number of microcultures to be set up and finally on the amount of blood that will be required from patients. We thus explored whether the number of washes could be decreased from 10 to 5 washes without affecting, purity of sorted cells.

Yields were estimated by comparing the absolute number of specific T cells in the population before the sort (as measured by HLA-p tetramer staining) and the number of rosettes (Clinimers-coated T lymphocytes) obtained after the sort. These rosetted T cells are enumerated through two manual counts performed on two independent samples.

Purity of sorted cells was evaluated by HLA-p tetramer staining after amplification on feeder cells (Figure 4(b)).

As shown in Figures 4(c) and 4(d), the yields of sorted cells were improved by reducing the number of washes from 10 to 5 washes, without affecting the purity of antigen-specific T cells after amplification.

### 3.3. Step 3: Elimination of Magnetic Beads after an 8-Day Culture Period

**Figure 5:**
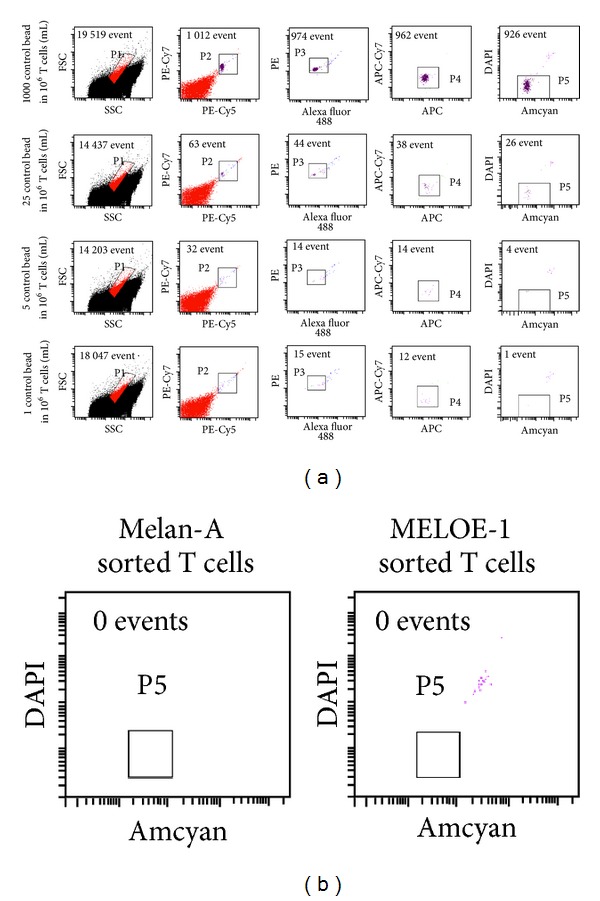
Detection of residual beads in amplified T cells. (a) Limit of detection of magnetic beads among a T cell population. Variable numbers of beads were mixed with 10^6^ T cells in 1 mL. Beads were detected by their autofluorescence in the various channels of a Facs Canto II. (b) Absence of residual beads in amplified T cells. After 8 days of culture of sorted T cells, each T cell suspension is placed inside a magnet to remove magnetic beads. After two rounds of elimination, the absence of residual beads is checked by flow cytometry.

During their polyclonal activation, sorted T cells downregulate their TCR surface expression and undergo many cell divisions. This results in rapid detachment of the Clinimers from T cells after 2 to 3 days in culture. Because the clinical grade magnetic beads used in Clinimers are not approved for injection to patients but only as an ancillary product in a cell production process, residual beads have to be removed from cell cultures, and the absence of beads has to be documented before adoptive cell transfer. This removal is performed after 8 days of amplification on cell suspensions adjusted to a concentration of 10^6^ cells/mL in 50 mL tubes that are placed inside a circular magnet for 10 minutes. A second round of bead elimination is performed in the same conditions. A sample of each cell suspension (corresponding to 1/100 of the final volume, that is, about 100 mL at this stage) is analyzed by flow cytometry to confirm the absence of residual beads. The beads are detected by their properties of autofluorescence in each detection channel of a Facs Canto II, allowing an accurate discrimination between beads and cell debris (Figure 5(a)). As illustrated, this detection method allows the detection of as little as one bead within 1 mL of a cell suspension containing 10^6^ T cells. As shown in Figure 5(b), the two rounds of magnet depletion were very efficient in removing all the residual beads from cell cultures.

### 3.4. Step 4: Amplification of Specific T Cells on Irradiated Allogeneic Feeder Cells

The last parameter that was evaluated was the average amplification factor of the sorted cells on irradiated feeder cells in order to calculate the initial number of PBMC needed to recover sufficient numbers of specific T cells at the end of the process. In our future clinical trial, we want to ensure the recovery of at least 10^8^ Melan-A- and MELOE-1-specific T cells, that is, at least 2 × 10^8^ tumor-specific T cells for injection to the patients. This minimal number of 2 × 10^8^ antigen-specific T cells to be infused to melanoma patients was chosen after analysis of the amounts of infused T cells in previous clinical trials of adoptive transfer [7, 24, 25]. For example, in our previous clinical trial of adoptive transfer of Melan-A specific T cell clones, patients received between 1.4 × 10^8^ and 2 × 10^9^ clonal T cells in a single injection [5]. However, the number of infused T cells did not seem to correlate with clinical benefit since among the two patients who experienced complete responses one received 2 × 10^8^ Melan-A-specific clonal T cells and the other 2 × 10^9^ clonal cells [5]. 

In the present study, as shown in Table 1, the amplification factors following the 14-day postsort cultures were of 1800 ± 432 for Melan-A-specific T cells and 2164 ± 923 for MELOE-1-specific ones. For Melan-A-specific T cells, this corresponded to about 10 to 11 cell division, and to about 8 to 12 cell divisions for MELOE-1-specific T cells. Setting the minimal amplification factor at 1000 for both specificities, to take a safety margin, it will be necessary to recover 10^5^ specific T lymphocytes after the sort. Considering a minimal sorting yield of 20%, the minimum number of specific cells to be sorted will be set at 5 × 10^5^ Melan-A- and MELOE-1-specific T lymphocytes.

The final amplification procedure will be thus performed starting from 10^5^ rosetted T cells, amplified on feeder cells in one 96-well plate, with 150 *μ*L per well of complete medium (15 mL per plate), containing 150 U/mL of IL-2 and 1 *μ*g/mL of PHA-L. After 8 days, lymphocytes are pooled and counted for a second amplification step, performed in 6-well plates. 

### 3.5. Control of the Specificity and the Reactivity of the Amplified T Cells

**Figure 6:**
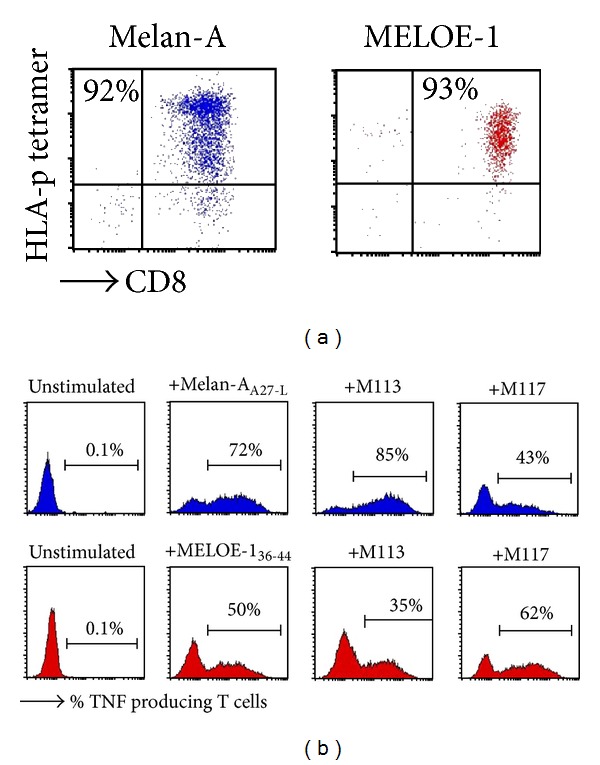
Purity and reactivity of sorted and amplified T cells. (a) Purity of sorted and amplified T cell populations is assessed by double staining with an anti-CD8 specific mAb and each specific HLA-p tetramers. (b) Activity of sorted T cells is measured by TNF production in response to the cognate peptide or to HLA-A2 melanoma cell lines expressing Melan-A and MELOE-1 antigens. After activation, T cells are stained with their specific HLA-p tetramer, and TNF producing cells are visualized by intracellular staining with an anti-TNF mAb. Blue histograms represent Melan-A- specific T cells and red ones MELOE-1-specific T cells.

Purity of sorted and amplified T cells was evaluated by HLA-p tetramer/CD8 double staining. As shown in Figure 6(a), Melan-A- and MELOE-1-specific T cell populations were at least 90% pure after the sorting and amplification steps. Activity of sorted populations is validated both on specific peptides and on HLA-A2 melanoma cells expressing Melan-A and MELOE-1 antigens by the % of tetramer positive cells producing TNF-*α* upon activation. As shown in Figure 6(b), both Melan-A (upper panel) and MELOE-1 (lower panel) sorted and amplified T cells are reactive against their cognate peptide and against melanoma cell lines expressing Melan-A and MELOE-1 antigens. Assessing the reactivity of Melan-A sorted T cells on melanoma cells was especially important, to document the activity of sorted cells in response to the naturally processed Melan-A epitope, as these T cells have been stimulated with a modified HLA-peptide. Indeed, the use of altered peptide ligands (ALP), which contain single amino acid substitutions that improve the affinity of the peptides for the HLA peptide-binding site, in immunotherapy is still a matter of debate. Some studies reported a suboptimal recognition of naturally processed epitopes by T cells stimulated by ALP either *in vitro* [26] or *in vivo* after vaccination [27]. In contrast, we and others previously documented that T cell repertoire specific for the Melan-A natural epitope and its modified counterpart were overlapping and that T cells primed with the modified analog were reactive against the natural epitope and against HLA-A2 melanoma cell lines [5, 6, 19, 28]. Reactivity of Melan-A sorted T cells on melanoma cell lines confirmed that stimulation with this analog peptide will not impair their antitumor activity and will validate their use in adoptive cell transfer.

### 3.6. Validation of the Whole Procedure in Three Blank Runs

**Figure 7:**
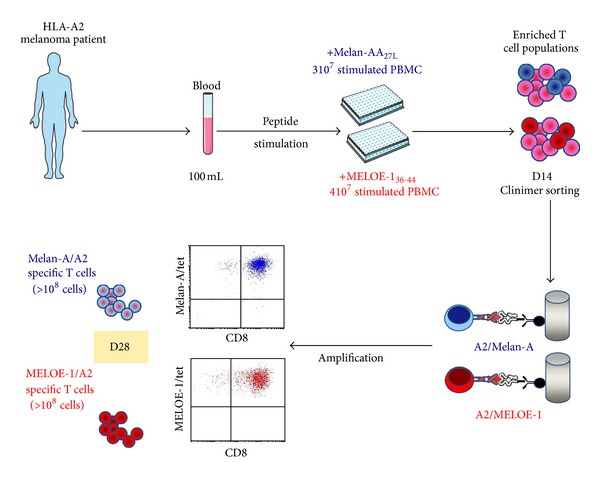
Design of the whole GMP process. At day 0, 100 mL of blood is collected from HLA-A2 melanoma patients. Total PBMC are stimulated with the two antigenic peptides during 14 days. At day 14, antigen-specific T cells are sorted using Clinimers, and 10^5^ rosetted T cells are seeded for amplification on feeder cells. At day 28, purity of amplified cells is assessed by flow cytometry and between 10^8^ and 5 × 10^8^ T lymphocytes specific for each peptide will be infused to the autologous melanoma patient.

Finally, the whole procedure (Figure 7) was validated in three blank runs performed in the Unit of Cell Therapy, in GMP conditions. For these runs, 3 × 10^7^ and 4 × 10^7^ PBMC from metastatic HLA-A2 melanoma patients were, respectively, stimulated with Melan-A_A27L_ and MELOE-1_36-44_ peptides, in 144 or 192 microcultures. At the end of the peptide stimulation step, 5 × 10^6^ or 10^7^ T cells containing between 9 and 38% of specific T lymphocytes were sorted with Clinimers, with sorting yields ranging between 19 and 60% (except one with 11% due to a technical mishap) (Table 2).

This resulted in the recovery of at least 1.2 × 10^5^ antigen-specific T cells prior to the amplification step. After a 14-day amplification period, on feeder cells, in RPMI medium supplemented with 8% HS, 150 U/mL of IL-2, and 1 *μ*g/mL of PHA-L, specific T cells performed between 10 and 16 divisions, which led to the production of at least 9.2 × 10^8^ specific T lymphocytes. These amplification factors are higher than those obtained in preclinical assays, probably due to variability between human serum batches. Each specific T cell population was pure (>90% of specific T cells) and reactive against the cognate peptide and against HLA-A2 melanoma cell lines expressing Melan-A and MELOE-1 antigens.

### 3.7. Production Conditions, Release Criteria, and Administration of Therapeutic T Cells

The cell production process (Figure 7) should be conducted in a unit of cell therapy authorized by national health agencies (in our case, a unit of cell therapy of a university hospital). Furthermore, administration to the patient should be performed in a dedicated medical unit (in our case the Oncodermatology Unit of the Nantes hospital). On the day of injection, Melan-A- and MELOE-1-specific T cells are enumerated, and their specificity and activity against each cognate peptide are assessed. The amount of T cells (at least 10^8^), their purity (at least 90% of tetramer positive cells), and their activity (at least 50% of TNF-*α* producing T cells among tetramer positive ones) are release criteria. The former criteria of 50% of TNF-*α* producing T cells among tetramer positive ones take into account the heterogeneity in the activation status of such a polyclonal population.

To fulfill these criteria, we set specifications for each production step, detailed in Table 3. After the peptide stimulation step, the minimal amount of specific T cells should be at least 0.5 × 10^6^ specific T cells among 10^7^ total T cells, to ensure the recovery of at least 10^5^ specific T cells after the sorting step. Clinimers are prepared immediately before used in coating the HLA-peptide monomers on antibody-coated beads. The coating efficiency is checked by flow cytometry using an HLA specific antibody, and the minimal RFI obtained should be of 20 or greater. After the step of bead removal, the total absence of residual beads is assessed using the method developed in Step 3. Finally, microbiological controls performed by an independent laboratory ensure sterility of the final product.

If each T cell population meets these criteria, T lymphocytes are pooled and adjusted at a concentration ranging between 10^6^ and 5 × 10^6^ cells/mL in a volume of 200 mL of a pharmaceutical solution of 4% human albumin, in a bag which is a medical device. This cell preparation remains stable in terms of purity and activity at least 2 hours. The delay between the preparation of the cell suspension and its injection should be less than two hours. The product is then administered intravenously at a rate of 3 mL/min under medical supervision.

## 4. Conclusion

In conclusion, our results demonstrate the many advantages of our GMP procedure for the production of therapeutic melanoma-specific T lymphocytes. (i) From a practical point of view, the initial blood sample volume is still reasonable and can be obtained from most patients. (ii) The two selected antigenic peptides allow the expansion of specific T cells in the vast majority of HLA-A2 patients, and these T cells are reactive against melanoma cells. (iii) The short duration of the whole procedure results in the production of pure and polyclonal specific T cells that underwent a limited number of divisions and should thus persist *in vivo* after injection.

The therapeutic potential of the melanoma specific T cells obtained with this procedure will soon be evaluated in an upcoming clinical trial supported by the French National Cancer Institute (INCa) that will include 17 metastatic melanoma patients.

## Figures and Tables

**Table 1:** Amplification rates of antigen-specific T cells on irradiated allogeneic feeder cells.

Peptide	Number of sorted specific cells	Final number of amplified specific cells	Amplification factor^1^	Average number of cell divisions
Melan-A_A27L_	10^5^	1.16 × 10^9^	1160	10
	8 × 10^4^	2.46 × 10^8^	3075	11
	5 × 10^3^	4.3 × 10^6^	860	10
	5 × 10^3^	1.3 × 10^7^	2600	11
	10^5^	1.38 × 10^9^	1380	10
			1815 ± 432	10.4 ± 0.5

MELOE-1_36-44_	3 × 10^5^	6 × 10^8^	2000	11
	10^5^	1.97 × 10^8^	1970	11
	8 × 10^4^	2.6 × 10^6^	325	8
	2.4 × 10^4^	1.35 × 10^8^	5625	12
	2 × 10^4^	1.8 × 10^7^	900	10
			2164 ± 923	10.4 ± 1.5

^1^Amplification factors are estimated from the final number of amplified T cells divided by the number of rosetted T cells seeded on feeder cells.

**Table 2:** Validation runs for the selection and amplification of Melan-A- and MELOE-1- specific T lymphocytes from HLA-A2 melanoma patient-derived PBMC.

Run ID	*≠*1	*≠*2	*≠*3	*≠*1	*≠*2	*≠*3
Peptide	Melan-A_A27L_			MELOE-1_36-44_		
Number of stimulated PBMC	3 × 10^7^	3 × 10^7^	3 × 10^7^	4 × 10^7^	4 × 10^7^	4 × 10^7^

Number of sorted cells and fraction of specific T cells	10^7^	5 × 10^6^	5 × 10^6^	5 × 10^6^	5 × 10^6^	10^7^
	(9.3%)	(34%)	(38%)	(11%)	(16%)	(9%)
Theoretical number of specific T cells^1^	9.3 × 10^5^	1.7 × 10^6^	1.9 × 10^6^	5.5 × 10^5^	8 × 10^5^	9 × 10^5^
Recovered number of sorted specific T cells	3.9 × 10^5^	6.6 × 10^5^	2.1 × 10^5^	1.2 × 10^5^	4.8 × 10^5^	1.7 × 10^5^
Sorting yield^2^	42%	39%	11%	22%	60%	19%
Final number of amplified specific T cells and amplification yield^3^	1.7 × 10^10^	6.9 × 10^9^	1.3 × 10^10^	2 × 10^9^	9.2 × 10^8^	4.4 × 10^9^
	(×43500)	(×10000)	(×62000)	(×16700)	(×1900)	(×25900)
Average number of cell divisions	15	13	16	14	10	15
Purity of amplified T cells^4^	95%	99%	92%	90%	91%	93%
Activity of amplified T cells on the cognate peptide^5^	82%	71%	72%	75%	53%	50%

^1^The theoretical number of specific cells is estimated from the % of tetramer-positive cells in the sorted population. ^2^Sorting yields are calculated by dividing the number of rosetted T cells after sorting by the theoretical number of specific T cells in the sorted populations. ^3^Amplification factors are estimated from the final number of amplified T cells divided by the number of rosetted T cells seeded for amplification on feeder cells. ^4^Purity of sorted and amplified T cells is assessed by tetramer/CD8 double staining. ^5^% of TNF producing T cells among tetramer positive cells : reactivity of amplified T cells is assessed by tetramer/TNF double staining, after peptide activation.

**Table 3:** Specifications and release criteria for manufactured T cell products.

Production steps	Specifications/release criteria
Peptide stimulation step	≥0.5 × 10^6^ specific T cells among a maximal number of 10^7^ total T cells
Coating of HLA-p on Chim-AvT beads	RFI ≥ 20 assessed by HLA labeling
Sorting step	≥10^5^ rosetted T cells
Amplification step	≥10^8^ specific T cells (RC)^1^

Quality controls	

Purity	≥90% purity assessed by tetramer labeling (RC)
Activity	≥50% TNF producing T cells among tetramer positive ones (RC)
Safety controls	Absence of microbiological contamination (RC)

^1^RC: release criteria.
